# Impact of Tiered Service Delivery Model on Antifibrotic Adherence and Outcomes Among Patients With Interstitial Lung Disease

**DOI:** 10.1111/crj.70239

**Published:** 2026-10-25

**Authors:** Fred Fyles, Alice McKinnon, Sarah Agnew, Joanne Sutcliffe, Sarah Brooks, Jacqueline Barnett, Siobhan McGarvie, Annelise Simons, Apollo Kwok, Vernoica Yioe, Lisa G. Spencer

**Affiliations:** ^1^ Liverpool Interstitial Lung Disease Service Aintree Hospital, Liverpool University Hospitals NHS Foundation Trust Liverpool UK; ^2^ University of Liverpool Liverpool UK

**Keywords:** antifibrotic agents, delivery of health care, interstitial lung diseases, mortality, treatment outcomes

## Abstract

**Introduction:**

Within England, Interstitial Lung Disease (ILD) services are in the process of adopting tiered ‘shared‐care’ service delivery to address increased demand. There are currently no data evaluating this model. We report on clinical outcomes of our Merseyside shared‐care ILD service delivery over 2 years.

**Methods:**

We conducted a retrospective observational study of adults with ILD started on antifibrotic therapy through our regional centre from 01/01/2022 to 31/12/2023. Alongside demographics and clinical data, we reviewed mortality and antifibrotic adherence. Patients were grouped on tier status of follow‐up site: Tier 3 were Specialist ILD Network Hubs, Tier 2 were specialist centres with ILD nurse access, and Tier 1 were general respiratory centres with remote support from Tier 3 ILD nurses.

**Results:**

A total of 275 patients were commenced on antifibrotic therapy (mean age 72.3, 69% male, 68% on nintedanib), with comparable cohorts across tiers. Twelve‐month all‐cause antifibrotic discontinuation was 40%, and was associated with increased age (HR 1.04, *p* = 0.001), but not with tier status of follow‐up site (*p* = 0.084). Twelve‐month survival was 79%, and was not significantly different between tiers (*p* = 0.3). Progressive fibrotic ILD was associated with increased mortality risk, whereas higher BMI and baseline lung function were associated with reduced mortality. Patients on pirfenidone were more likely to have intolerable side effects (HR 1.60, *p* = 0.029), and patients stopping antifibrotics had a significantly increased mortality risk (adjusted HR 2.18, *p* = 0.003).

**Conclusion:**

This retrospective real‐world study suggests that a tiered approach to ILD service delivery—provided non‐specialist sites are given appropriate support—can be a safe and effective model.

## Graphical abstract

In the United Kingdom there is a move towards shared‐care models for interstitial lung disease, but outcomes from these models are underexplored. We reviewed 2 years of antifibrotic prescribing and follow‐up across our tiered service and found no difference in antifibrotic adherence or mortality based on whether someone was at a specialist or non‐specialist centre.

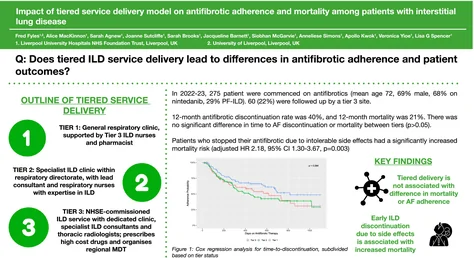

## 1 Introduction

Interstitial lung disease (ILD) describes a heterogenous group of conditions characterised by inflammation and/or fibrosis to the lung parenchyma, with associated gas exchange impairment, leading to respiratory symptoms, lung function decline, and mortality [1]. Idiopathic Pulmonary Fibrosis (IPF) is the most commonly encountered clinical entity, with incidence around 8000–9000 cases in the United Kingdom each year [2], and a poor estimated 5‐year survival of 40% [3]. Other ILDs can also develop a progressive phenotype referred to as progressive fibrosing ILD (PF‐ILD) [4].

In the United Kingdom, two antifibrotic (AF) medications are available for IPF: pirfenidone, a synthetic piridone demonstrated to slow lung function decline [5, 6]; and nintedanib, a tyrosine kinase inhibitor that has also demonstrated a reduction in lung function decline [7, 8, 9]. Evidence supporting nintedanib has extended across a broader range of progressive‐fibrosing ILDs, such as systemic sclerosis‐associated ILD (SSc‐ILD), with evidence suggesting no heterogeneity in treatment effects across a range of SSc‐ILD subgroups [10].

Both are associated with adverse events (AEs), with adherence presenting a challenge for patients and clinicians: within landmark randomised controlled trials (RCTs), the majority of participants experienced AEs, with discontinuation rates ranging from 14.4% to 25.9% [5, 6, 7, 8, 9]. Similarly, real‐world experience in ILD subtypes, including SSc‐ILD, has highlighted the importance of treatment tolerability, with gastrointestinal (GI) side effects and the need for dosing reductions reported [11]. Various strategies to encourage adherence have been proposed, including the use of medication to manage AEs and patient support programmes [12].

Within the United Kingdom, ILD services providing access to antifibrotic drugs for patients have faced challenges due to increased demand, with an increase in wait times from referral to clinic presentation over the past decade [13]. A recently published guideline for ILD care in England has recommended a ‘transformative’ Integrated Care Pathway for ILD services, restructuring ILD services into regional tiered networks, supported by a Specialised ILD Network Hub, to ensure ‘all patients have access to a multidisciplinary disease‐specific management plan with care taking place closer to home’ [14]. At present, however, there are minimal data on whether tiered service delivery is associated with differences in outcomes.

Within the Merseyside region in North‐West England, the Merseyside ILD Network has operated a shared care model with tiered service delivery for a number of years. Aintree University Hospital acts as a ‘Tier 3’ regional specialist hub, with a National Health Service England‐commissioned specialist ILD service that coordinates the regional multi‐disciplinary team (MDT) meeting, prescribes antifibrotic therapy, and provides specialist support to other centres within the network. Tier 2 sites provide dedicated local ILD clinics, whereas Tier 1 sites deliver ILD care within a general respiratory clinic setting. Both Tier 1 and Tier 2 sites are supported remotely by the Tier 3 hub, which was able to offer advice on aspects of ILD care such as side effect management and antifibrotic dose adjustment.

In anticipation of proposed changes to ILD service delivery in England, we report the clinical outcomes of shared care and tiered service delivery over a 2‐year period within our patient cohort.

## 2 Methods and Materials

We conducted a retrospective observational cohort study at Aintree University Hospital (Liverpool, UK), a regional ILD centre within the North‐West of England. Adult (aged ≥ 18 years) patients with physician‐confirmed ILD who were commenced on AF medications (nintedanib or pirfenidone) through the regional MDT from 01/01/2022 to 31/12/2023 were included. Patients who transferred to our service after starting AF elsewhere, or patients who moved out of the area after commencing AF, were excluded from analysis.

We collected baseline data including demographics (age, sex, body mass index [BMI], ILD subtype, smoking history) and spirometric data at AF start (Forced Expiratory Volume in 1 Second [FEV_1_], % predicted FEV_1_ [FEV1%P], Forced Vital Capacity [FVC], % Predicted FVC [FVC%P], FEV1/FVC, and % Predicted Transfer Factor of the Lung for Carbon Monoxide [TLCO%P]). Data on comorbidities were collected, and the burden of comorbidity was assessed using the Charlson Comorbidity Index (CCI), a weighted score of comorbidities developed to aid mortality prediction [15]. Data were collected on AF discontinuation—including time on AF, reason, and whether patient‐initiated or physician‐initiated—and mortality up until 01/01/2025.

Patients were grouped based on tier status of parent site responsible for routine clinical follow‐up, using definitions in line with the Action for Pulmonary Fibrosis ILD care pathway—see Table S1. Tier 3 represented the National Health Service England (NHSE) commissioned regional ILD hub, with specialist ILD physicians and nurses, thoracic radiology and pathology input, responsibility for the regional MDT, and antifibrotic prescribing rights. Tier 2 sites provided dedicated local ILD clinics run by respiratory physicians and nurses with ILD expertise, and a local MDT. Tier 1 sites provided ILD follow‐up within general respiratory clinics, without a dedicated MDT. Tier 1 and Tier 2 sites were able to access specialist support from the Tier 3 site, including clinical and pharmacy advice. Throughout the study period, the Tier 3 site prescribed antifibrotic therapy, whereas Tier 1 and Tier 2 sites undertook routine clinical follow‐up and blood monitoring. Specialist ILD nursing and pharmacy support was available by telephone and email, including advice regarding treatment‐related side effects, abnormal monitoring results, and antifibrotic dose interruption. Patients were typically followed up at their geographically closest site, with some exceptions made based on patient preference.

### 2.1 Outcomes

Our primary outcome was all‐cause AF discontinuation, expressed as time‐to‐discontinuation. Secondary outcomes included patient‐initiated AF discontinuation due to intolerable side effects, reasons for physician‐initiated or patient‐initiated AF discontinuation, and all‐cause mortality. Tier status, AF choice, and underlying disease (IPF vs. PF‐ILD) were used as comparative groups within our analysis.

### 2.2 Statistical Analysis

Baseline clinical characteristics were summarised for the entire cohort, and sub‐grouped by AF choice, ILD subtype, and tier status using mean (μ) ± standard deviation (SD) or median and interquartile range (IQR) for continuous variables where appropriate, and frequency/percentage for categorical variables. Wilcoxon rank‐sum test and Kruskal–Wallis test were used to compare 2 and > 2 groups respectively, and analysis of variance (ANOVA) was used to compare continuous variables between multiple groups, with Bonferroni correction used for multiple pair‐wise comparisons. Chi‐squared testing was used to compare categorical variables, with use of Yates' continuity correction where appropriate (*n* < 10).

Kaplan–Meier survival curves and univariable Cox proportional‐hazards regression were used to compare time to all‐cause AF discontinuation and all‐cause mortality. To assess whether associations between patient tier and outcomes were not affected by potential differences in patient characteristics, complete‐case multivariable Cox sensitivity analyses were performed, adjusting for age, sex, CCI, ILD subtype and baseline spirometry. Patients who, as of 01/01/2025, remained adherent to AF (in the case of time to AF discontinuation) or alive (in the case of mortality) were censored. Patients who died whilst still on AF medication were censored for the purpose of time to AF discontinuation analysis. All analyses were carried out using RStudio Version 2024.04.2 + 764 (2024.04.2 + 764) Copyright (C) 2024 by Posit Software, PBC.

## 3 Results

### 3.1 Demographics and Patient Cohort

From 01/01/2022 to 31/12/2023, 275 patients were commenced on AF therapy through our ILD centre. The mean age was 72.3, and 69% were male. A total of 186 patients, representing 68% of the cohort, were commenced on nintedanib, with 89 patients (32%) commenced on pirfenidone. Median wait time from referral to MDT discussion was 44 days (IQR: 31–64 days). A total of 60 patients (22%) were followed up by a Tier 3 site, with Tier 2 and Tier 1 sites following up 125 (45%) and 90 (33%) patients, respectively. Cohorts at different tiers were comparable, with no significant differences in baseline demographics, comorbidities, MDT wait time, or spirometric data. Similarly there was no significant difference in variables on individual pair‐wise comparison of tiers, after Bonferroni correction for multiple comparisons. Full baseline demographics are available in Table 1.

**TABLE 1:** Overall cohort of patients with interstitial lung disease, started on antifibrotic therapy 01/01/2022–31/12/2023, subdivided by tier.

		Total (*n* = 275)	Tier 1 (*n* = 90)	Tier 2 (*n* = 125)	Tier 3 (*n* = 60)	*p*
Age at AF start, years (μ ± SD)		72.3 ± 8.7	72.4 ± 9.4	72.9 ± 7.9	70.9 ± 9.1	0.299
Sex, *n* (%)	Male	191 (69%)	64 (71%)	91 (73%)	36 (60%)	0.191
	Female	84 (31%)	26 (29%)	34 (27%)	24 (40%)	
BMI at AF start, kg/m^2^ (μ ± SD)		28.4 ± 5.3	28.5 ± 5.2	27.7 ± 5.0	29.5 ± 5.8	0.212
Smoking history, *n* (%)	Current smoker	6 (2%)	1 (1%)	3 (2%)	2 (3%)	0.770
	Ex‐smoker	160 (58%)	50 (56%)	76 (61%)	34 (57%)	
	Never smoker	101 (37%)	37 (41%)	43 (34%)	21 (35%)	
	UNK	8 (3%)	2 (2%)	3 (2%)	3 (5%)	
Pack year history among smokers/ex‐smokers (μ ± SD)		23.3 ± 20.1	21.3 ± 15.9	22.8 ± 20.8	27.8 ± 24.0	0.535
Antifibrotic choice	Nintedanib	186 (68%)	66 (73%)	80 (64%)	40 (67%)	0.347
	Pirfenidone	89 (32%)	24 (27%)	45 (36%)	20 (33%)	
ILD subtype, *n* (%)	IPF, FVC%*p* ≥ 80, *n* (%)	23 (8%)	9 (10%)	12 (10%)	2 (3%)	0.464
	IPF, FVC%*p* < 80, *n* (%)	173 (63%)	51 (57%)	81 (65%)	41 (68%)	
	PF‐ILD, *n* (%)	79 (29%)	30 (33%)	32 (25%)	17 (28%)	
MDT wait time, days (median, IQR)		44, 31–64	45, 35–70	42, 28–58	52, 35–86	0.052
Charlson comorbidity index (μ ± SD)		4.6 ± 1.5	4.6 ± 1.6	4.7 ± 1.4	4.4 ± 1.4	0.271
FEV_1_, raw, L (μ ± SD)		1.9 ± 0.6	1.9 ± 0.5	2.0 ± 0.6	1.9 ± 0.6	0.685
FEV_1_, % predicted, % (μ ± SD)		74.4 ± 14.9	74 ± 15	76 ± 14	72 ± 16	0.292
FVC, raw, L (μ ± SD)		2.3 ± 0.6	2.3 ± 0.6	2.3 ± 0.6	2.3 ± 0.7	0.835
FVC, % predicted, % (μ ± SD)		69.2 ± 13.3	69 ± 14	71 ± 13	67 ± 13	0.289
FEV1/FVC ratio (μ ± SD)		83.3 ± 8.6	83.6 ± 7.5	83.6 ± 8.3	82.5 ± 10.4	0.482
TLCO, % predicted, % (μ ± SD)		44.0 ± 13.0	45 ± 13	44 ± 13	44 ± 13	0.867
First Antifibrotic, *n* (%)	Y	240 (87%)	83 (92%)	107 (86%)	50 (83%)	0.208
	N	35 (13%)	7 (8%)	18 (14%)	10 (17%)	

*Note:* *p*‐values for smoking history exclude patients with unknown smoking status. *p*‐value for ILD subtype compares IPF with PF‐ILD.

Overall, patients started on nintedanib were younger (70.9 ± 9.3 vs. 75.2 ± 6.4, *p* < 0.001), had a significantly lower CCI (4.5 ± 1.6 vs. 4.8 ± 1.1, *p* = 0.013), and were less likely to have previously trialled an AF drug (7% vs. 25%, *p* < 0.001) than those started on pirfenidone. There was a significant difference in ILD subtype between the pirfenidone and nintedanib groups, in line with the UK's National Health and Clinical Excellence (NICE) authorisation—see Table 2.

**TABLE 2:** Cohort of patients with ILD, started on antifibrotic therapy from 01/01/2022 to 31/12/2023, subdivided by antifibrotic choice.

		Nintedanib (*n* = 186)	Pirfenidone (*n* = 89)	*p*
Age at AF start, years (μ ± SD)		70.9 ± 9.3	75.2 ± 6.4	< 0.001
Sex, *n* (%)	Male	124 (67%)	67 (75%)	0.147
	Female	62 (33%)	22 (25%)	
BMI at AF start, kg/m^2^ (μ ± SD)		28.5 ± 5.3	28.0 ± 5.2	0.567
Smoking history, *n* (%)	Current smoker	3 (2%)	3 (3%)	0.243
	Ex‐smoker	104 (56%)	56 (63%)	
	Never smoker	74 (40%)	27 (30%)	
	UNK	5 (3%)	3 (3%)	
Pack year history among smokers/ex‐smokers (μ ± SD)		20.5 ± 17.6	28.7 ± 23.4	0.020
Tier status	Tier 1	66 (35%)	24 (27%)	0.347
	Tier 2	80 (43%)	45 (51%)	
	Tier 3	40 (22%)	20 (22%)	
ILD subtype, *n* (%)	IPF, FVC%P ≥ 80	23 (12%)	0	< 0.001
	IPF, FVC%P < 80	84 (45%)	89 (100%)	
	PF‐ILD	79 (42%)	0	
MDT wait time, days (median, IQR)		42, 31–58	47, 33–75	0.071
Charlson comorbidity index (μ ± SD)		4.5 ± 1.6	4.8 ± 1.1	0.013
FEV1, L (μ ± SD)		1.9 ± 0.6	2.0 ± 0.5	0.259
FEV1%P, % (μ ± SD)		73.5 ± 16.2	76.4 ± 11.6	0.071
FVC, L (μ ± SD)		2.3 ± 0.7	2.4 ± 0.5	0.286
FVC%P, % (μ ± SD)		69.2 ± 15.3	69.3 ± 7.5	0.847
FEV1/FVC ratio (μ ± SD)		83.5 ± 9.1	83.0 ± 7.4	0.309
TLCO%P, % (μ ± SD)		43.8 ± 12.9	44.3 ± 13.1	0.796
First antifibrotic, *n* (%)	Y	173 (93%)	67 (75%)	< 0.001
	N	13 (7%)	22 (25%)	

*Note:* *p*‐values for smoking history exclude patients with unknown smoking status. *p*‐value for ILD subtype compares IPF with PF‐ILD.

### 3.2 Overall Adherence

From AF initiation until death or end of follow‐up, 136 (49%) patients discontinued their AF therapy: 86 on nintedanib and 50 on pirfenidone, representing 46% and 56% of their initial cohorts respectively. Patients who discontinued AF were significantly older (74.1 ± 7.5 v 70.6 ± 9.4, *p* = 0.002), with a significantly higher CCI (4.7 ± 1.3 vs. 4.5 ± 1.7, *p* = 0.04), lower FVC (2.2 ± 0.6 vs. 2.4 ± 0.7, *p* = 0.035), and lower TLCO%P (42 ± 13 vs. 46 ± 13, *p* = 0.04). Other demographic and spirometric variables were not significantly different between the two groups—see Table 3. On Kaplan–Meier analysis, 12‐month all‐cause AF discontinuation was 40% (95% CI 34%–46%) and median time to discontinuation was 667 days (95% CI 472–818), see Figure 1.

**TABLE 3:** Patients with ILD, started on antifibrotic therapy 01/01/2022–31/12/2023, subdivided by whether they continued or discontinued antifibrotic (AF) therapy.

		Continued AF (*n* = 139)	Discontinued AF (*n* = 136)	*p*
Age at AF start, years (μ ± SD)		70.6 ± 9.4	74.1 ± 7.5	0.002
Sex, *n* (%)	Male	100 (72%)	91 (67%)	0.365
	Female	39 (28%)	45 (33%)	
BMI at AF start, kg/m^2^ (μ ± SD)		28.8 ± 5.0	27.9 ± 5.5	0.103
Smoking history, *n* (%)	Current smoker	3 (2%)	3 (2%)	0.999
	Ex‐smoker	79 (57%)	81 (60%)	
	Never smoker	50 (36%)	51 (38%)	
	UNK	7 (5%)	1 (1%)	
Pack year history among smokers/ex‐smokers (μ ± SD)		24.5 ± 21.9	22.1 ± 18.2	0.829
Antifibrotic choice, *n* (%)	Nintedanib	100 (72%)	86 (63%)	0.123
	Pirfenidone	39 (28%)	50 (37%)	
MDT wait time, days (median, IQR)		42, 29–62	47, 36–70	0.054
Charlson comorbidity index (μ ± SD)		4.5 ± 1.7	4.7 ± 1.3	0.044
FEV1, raw, L (μ ± SD)		2.0 ± 0.6	1.9 ± 0.5	0.253
FEV1, % predicted, % (μ ± SD)		73.3 ± 15.8	75.6 ± 13.8	0.364
FVC, raw, L (μ ± SD)		2.4 ± 0.7	2.2 ± 0.6	0.035
FVC, % predicted, % (μ ± SD)		68.7 ± 14.0	69.8 ± 12.6	0.812
FEV1/FVC ratio (μ ± SD)		83 ± 9	84 ± 8	0.349
TLCO, % predicted, % (μ ± SD)		46 ± 13	42 ± 13	0.041

*Note:* *p*‐values for smoking history exclude patients with unknown smoking status. *p*‐value for ILD subtype compares IPF with PF‐ILD.

**FIGURE 1:**
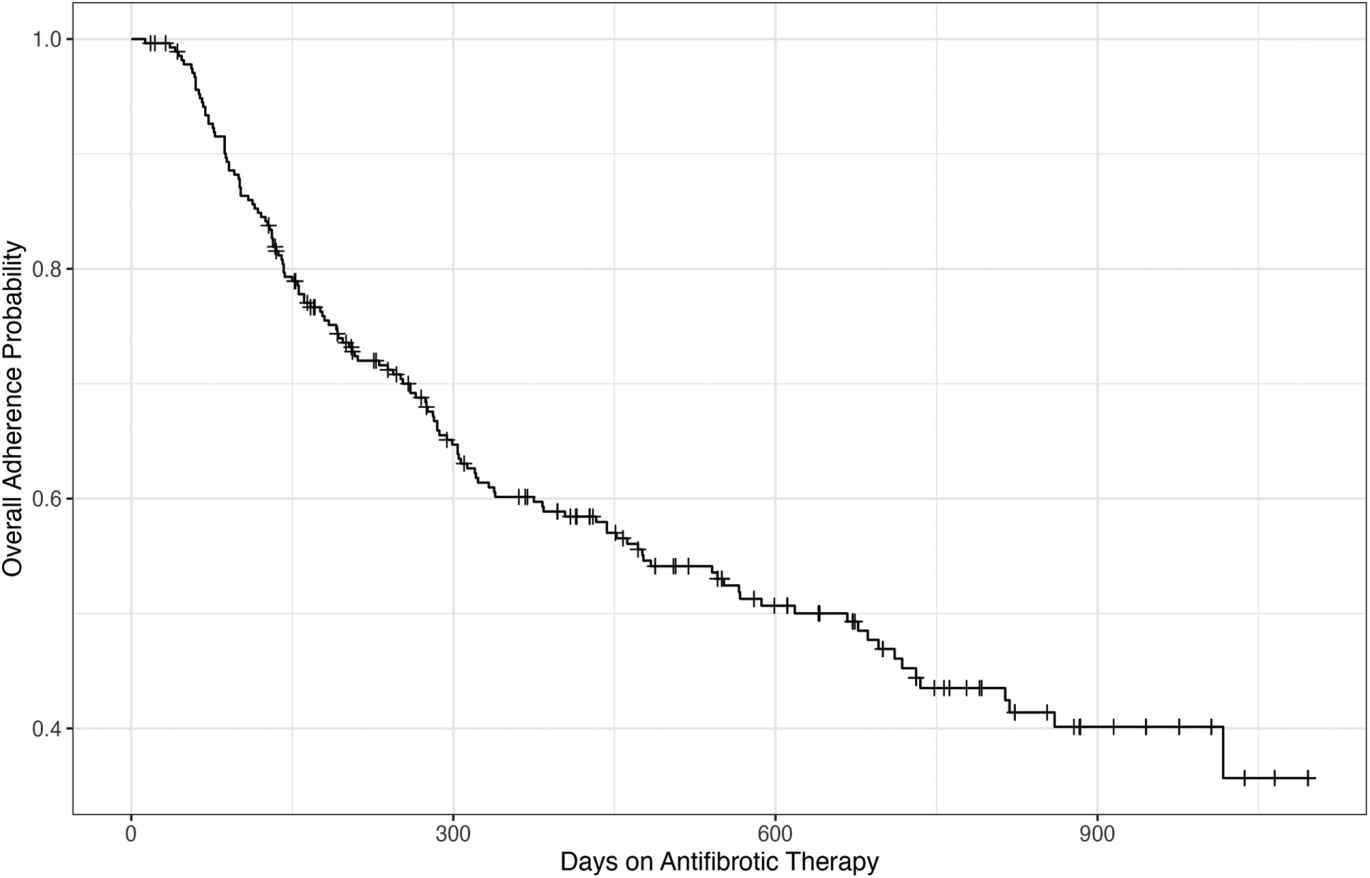
Kaplan–Meier analysis of time‐to discontinuation among patients with ILD started on antifibrotic therapy 01/01/2022‐31/12/2023 (*n* = 275).

All three tiers had similar 12‐month discontinuation rates (Tier 1: 32%, 95% CI 22%–42%; Tier 2: 46%, 95% CI 36%–54%; Tier 3: 40%, 95% CI 26%–51%), and on Cox regression modelling there was no significant difference in AF discontinuation rate between tiers (*p* = 0.084)—see Figure 2. On univariate Cox regression modelling, older age was associated with increased risk of discontinuation (HR 1.04, 95% CI 1.01–1.06, *p* = 0.001), whereas protective factors included higher baseline BMI (HR 0.96, 95% CI 0.93–1.00, *p* = 0.026) and higher baseline TLCO%P (HR 0.98, 95% CI 0.96–0.99, *p* = 0.005). Sex, AF choice, ILD subtype, CCI, and other spirometric measures were not associated with a significant difference in adherence. In multivariable analysis, adjusting for age, sex, CCI, ILD subtype, and baseline FVC% predicted (*n* = 270 complete cases) there was no evidence of an increased risk of antifibrotic discontinuation in Tier 2 (HR 1.04, 95% CI 0.68–1.60, *p* = 0.856) or Tier 1 (HR 0.65, 95% CI 0.40–1.06, *p* = 0.085) compared to Tier 3 patients.

**FIGURE 2:**
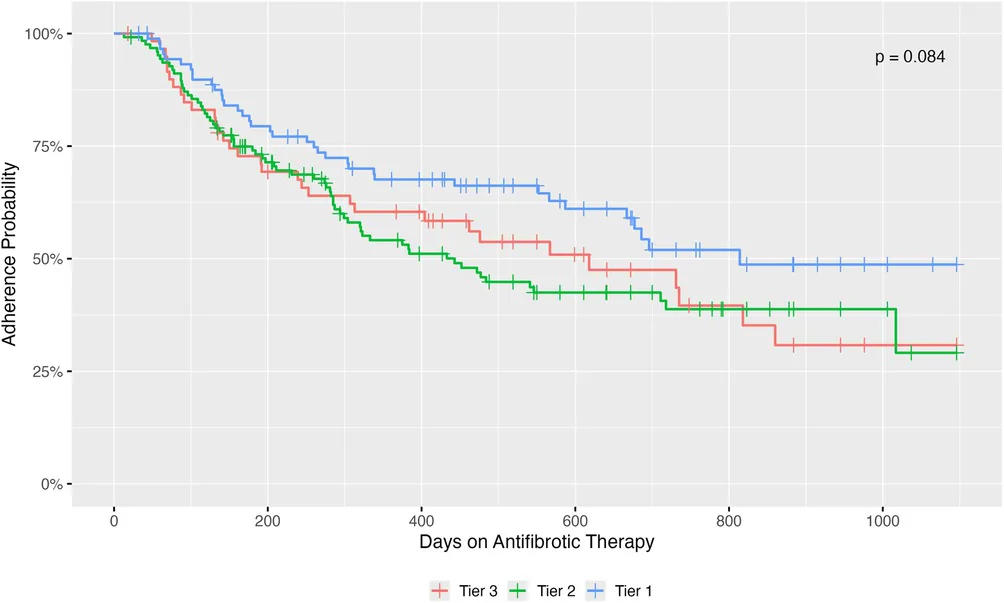
Kaplan–Meier analysis of time‐to‐antifibrotic discontinuation among patients with ILD started on antifibrotic therapy 01/01/2022–31/12/2023 stratified by tier status.

### 3.3 Patient‐Initiated Discontinuation

A total of 89 patients (52 on nintedanib and 37 on pirfenidone) self‐terminated their AF due to side effects, with the most common experienced being diarrhoea (*n* = 39), nausea/vomiting (*n* = 33) and appetite loss (*n* = 33). Seventy‐two percent of patients who self‐terminated their AF reported multiple side effects leading to their decision. Compared to those on nintedanib, patients taking pirfenidone were more likely to report intolerable appetite loss (19% vs. 9%, *p* = 0.028), lethargy/malaise (18% vs. 3%, *p* < 0.001) and dizziness (6% vs. 1%, *p* = 0.003); patients on nintedanib were more likely to report intolerable diarrhoea (18% vs. 6%, *p* = 0.005). See Table S2 for further detail on side effect profiles.

On Cox regression modelling, patients on pirfenidone were more likely to self‐terminate therapy, with a HR of 1.60 (95% CI 1.05–2.44, *p* = 0.029) compared to those on nintedanib. Older age was associated with an increased risk of patient‐initiated AF discontinuation (HR 1.04, 95% CI 1.01–1.07, *p* = 0.004), and higher TLCO%P was associated with a reduced risk (HR 0.98, 95% CI 0.96–0.99, *p* = 0.008). Other demographics, including sex, BMI, ILD subtype, CCI, and other spirometric measures, were not associated with increased risk of patient‐initiated AF discontinuation.

### 3.4 Mortality

Overall, 121 (44%) of patients died between AF commencement and 01/01/2025. Of these, 75 (62%) stopped their AF prior to death, and 46 (38%) continued their AF up until death. Among the entire cohort, 12‐month survival was estimated at 79% (95% CI 75%–84%) on Kaplan–Meier analysis—see Figure 3. There was no significant survival difference between tier status (*p* = 0.3), and on univariate Cox regression modelling, compared to follow‐up at a Tier 3 site, there was no increased mortality risk at Tier 2 (HR 1.02, 95% CI 0.66–1.60, *p* > 0.9) or Tier 1 sites (HR 0.74, 95% CI 0.45–1.22, *p* = 0.2)—see Figure 4. This was unchanged following adjustment for age, sex, CCI, ILD subtype, and baseline FVC% predicted (*n* = 270 complete cases), with no evidence of increased mortality in Tier 2 (HR 1.03, 95% CI 0.65–1.62, *p* = 0.913) or Tier 1 (HR 0.65, 95% CI 0.39–1.09, *p* = 0.103) compared to Tier 3 patients.

**FIGURE 3:**
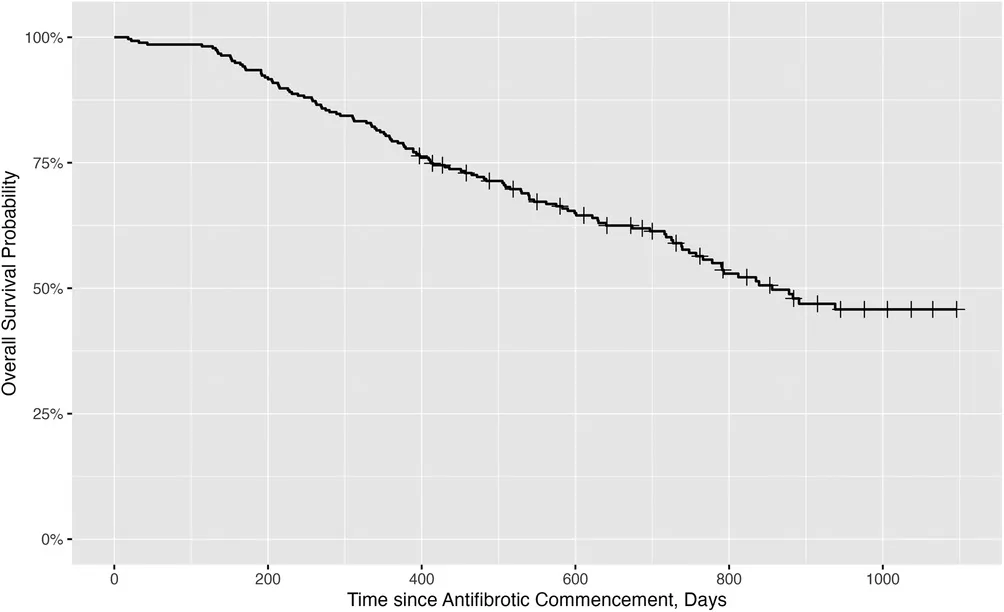
Kaplan–Meier analysis of mortality among patients with ILD started on antifibrotic therapy 01/01/2022‐31/12/2023 (*n* = 275).

**FIGURE 4:**
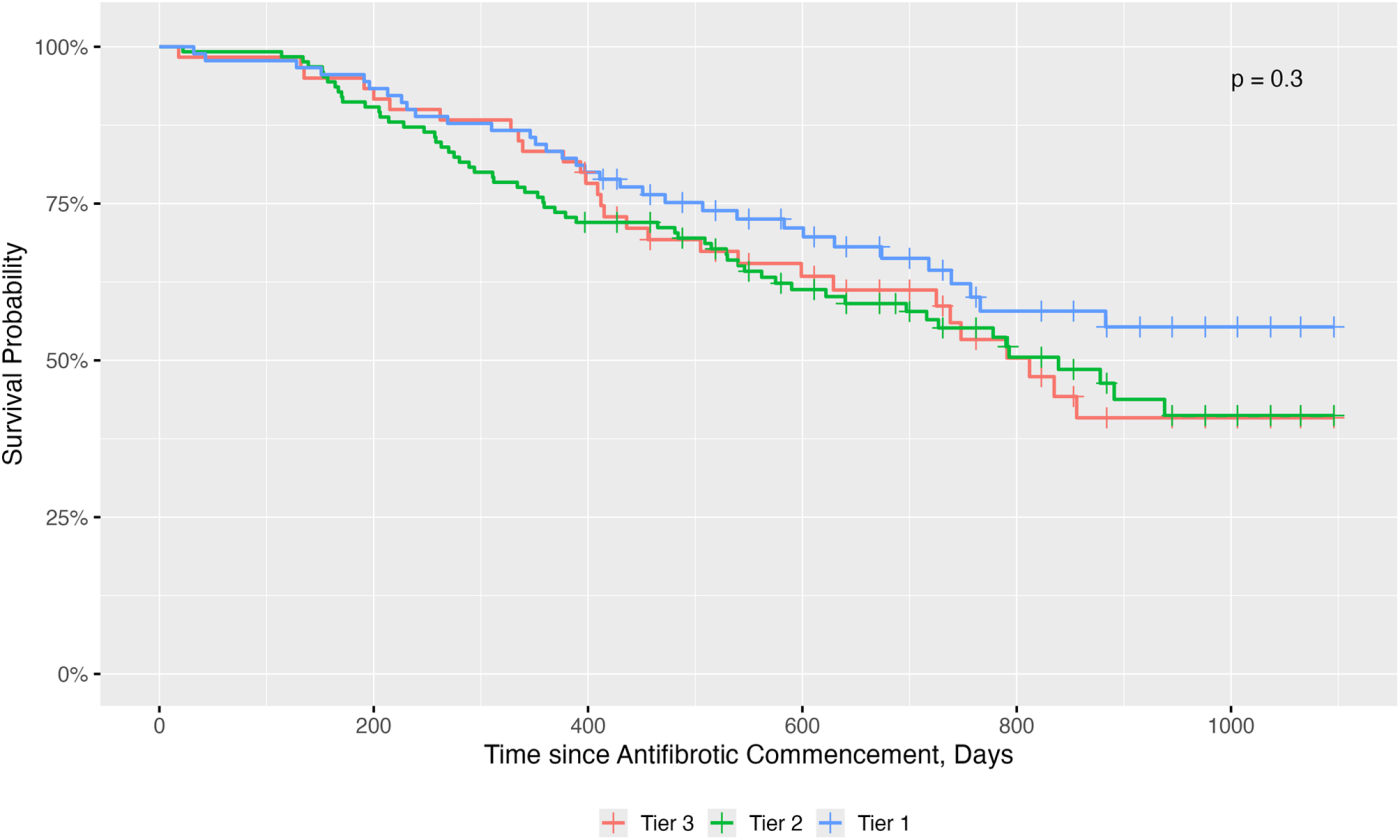
Kaplan–Meier analysis of mortality among patients with ILD, started on antifibrotic therapy 01/01/2022–31/12/2023, subdivided by tier status.

On univariate Cox regression modelling, the PF‐ILD phenotype was associated with an increased mortality risk (HR 1.58, 95% CI 1.09–2.30, *p* = 0.017), whereas higher BMI (HR 0.95, 95% CI 0.92–0.99, *p* = 0.012), baseline FVC%P (HR 0.98, 95% CI 0.97–1.0, *p* = 0.018), baseline FEV1%P (HR 0.99, 95% CI 0.98–1.00, *p* = 0.083), and baseline TLCO%P (HR 0.97, 95% CI 0.96–0.99, *p* = 0.002) were associated with a reduced mortality risk. Age, sex, CCI and AF choice were not associated with significant differences in mortality.

Patients who stopped their AF during follow‐up demonstrated a significantly increased mortality risk (*p* = 0.02)—see Figure 5. This finding persisted following adjustment for age, sex, BMI, CCI, ILD subtype and baseline spirometry results (adjusted HR 2.18, 95% CI 1.30–3.67; *p* = 0.003).

**FIGURE 5:**
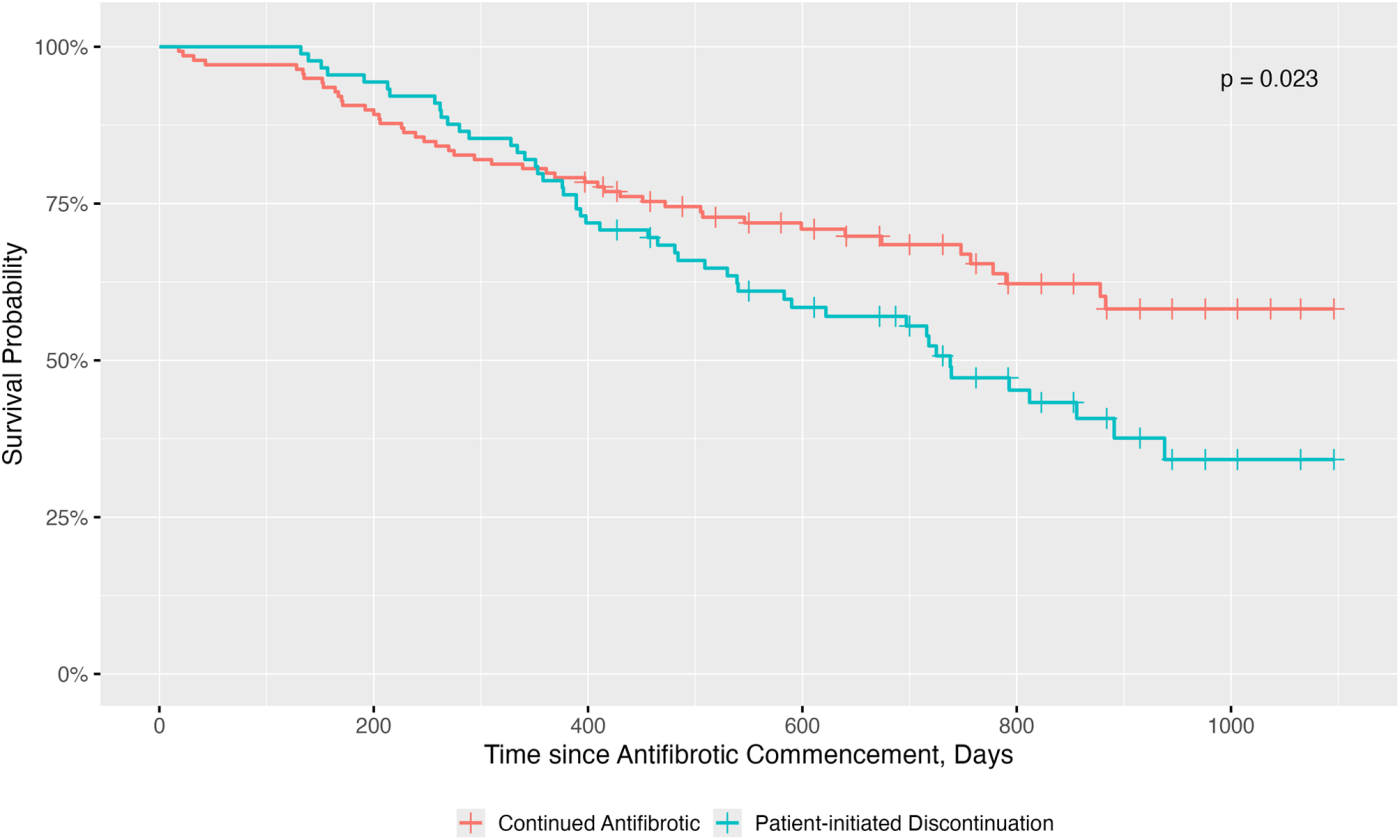
Kaplan–Meier analysis of mortality among patients with ILD according to patient‐initiated antifibrotic discontinuation.

## 4 Discussion

This study investigated the impact of a tiered service delivery system for ILD care on patient outcomes, including adherence to AF therapy and all‐cause mortality. We demonstrated that, among our cohort of patients initiated onto AF therapy by a Tier 3 site, subsequent shared care did not lead to an appreciable difference in patient outcomes. We also demonstrated several risk factors for all‐cause AF discontinuation, including increasing age, lower BMI and lower TLCO%P at AF initiation. AF choice appeared to impact side effect profile, with pirfenidone associated with increased rates of appetite loss, malaise and dizziness, and overall increased risk of patient‐initiated AF discontinuation. Our 12‐month survival was estimated at 79%, with lower BMI, lower baseline lung function and the PF‐ILD phenotype all being associated with increased mortality risk; importantly, patient‐led AF discontinuation was associated with a significantly increased mortality risk, highlighting both the importance of adherence support and that patients entering the later stages of life may not tolerate AF well.

To our knowledge, this is the first study to evaluate the impact of a decentralised model of ILD care in the United Kingdom on patient‐centred outcomes, a form of service delivery that is likely to become increasingly relevant in England following recommendations set out by Action for Pulmonary Fibrosis [14]. While our service has benefited from a number of years of experience in running a tiered service, these findings suggest that—provided appropriate and robust support is in place—non‐specialist sites may take over follow‐up of patients with ILD without an appreciable difference in outcomes. This may help provide confidence to other hubs to work towards a similar model. Our service has benefitted from good working relationships between colleagues, alongside working with colleagues outside the regional hub to develop ILD services locally.

Our study made use of a real‐world cohort, including patients with both IPF and PF‐ILD. Although our cohort was predominantly male (70%), this figure is slightly lower than typical ILD cohorts seen in RCTs [5, 6, 7, 8, 9], as well as UK registry data [13]. This is likely due to the inclusion of our PF‐ILD cohort, who typically have a more equal sex distribution, as demonstrated with the INBUILD study's patient cohort [9]. Our cohort's average age was 72.3 years, approximately in line with UK IPF registry data [16] and reflective of the increasing age of the UK ILD cohort over time [13].

We estimated an overall all‐cause discontinuation rate of 40% at 12 months, with the majority of discontinuation being patient‐led due to intolerable side effects. This figure is higher than that reported in the landmark RCTs, with IMPULSIS 1 and 2, ASCEND and INBUILD, all reporting 52‐week discontinuation rates of 14.4–25.9% [5, 6, 7, 8, 9]. However, numerous real‐world studies have demonstrated a markedly wider range of discontinuation rates than among study populations, with recent studies from European cohorts reporting 12‐month discontinuation rates of 33%–45.6% [17, 18, 19]. These findings of lower adherence in real‐world cohorts compared to RCT populations may be reflective of increased variability in care, heterogenous patient populations, and a less extensive support system outside of a study environment.

Our finding that increased age and worse baseline lung function are associated with a reduction in AF tolerability supports previous findings that have demonstrated both age [20, 21] and TLCO [22] can be used to predict discontinuation; although our study did not demonstrate that FVC was a marker for early discontinuation, as has been shown in other studies [23, 24], both FVC and TLCO can be used as proxy markers for ILD severity [22], which has been shown to affect AF adherence by Ogura et al. [25]. There have been suggestions that increasing age is associated with increased rates of AF‐related AEs [21], which may explain the higher rates of discontinuation among this cohort.

Our finding that reduced BMI was associated with both all‐cause AF discontinuation and all‐cause mortality has been demonstrated in numerous other real‐world studies [23, 26], and is an important consideration for those delivering ILD services. However, the fact that within our study BMI was not significantly associated with patient‐initiated AF discontinuation may suggest the relationship between low BMI and AF cessation is mediated by physician decision‐making—particularly where AF therapies may be stopped due to weight loss—or by known associations between low BMI and ILD progression/ILD‐related mortality [27], as opposed to increased risk of GI‐related side effects alone.

Our study benefits from a robust, well‐characterised, and large real‐world cohort, encompassing both IPF and PF‐ILD patients and reflective of current UK demographics in ILD care. Its conclusions are also strengthened by our prolonged follow period, which extends up to 24 months from AF initiation. The limitations of the study are similar to those common to any real‐world retrospective analyses, including missing data, which precluded the use of some parameters, such as longitudinal lung function data, exacerbations, hospitalisations, patient satisfaction and health‐related quality of life. Consequently, treatment‐related adverse events, particularly those that were mild or did not lead to treatment discontinuation, were likely to be under‐reported. Detailed side effect data were not available systematically for patients remaining on treatment; therefore, the adverse event profiles reported in this study should be interpreted as those contributing to treatment discontinuation rather than across the entire cohort. Similarly, given this was a retrospective analysis, patient‐reported outcome measures and data on patient satisfaction were not available. Although our findings suggested no appreciable difference in clinical outcomes, patient experience is an important measure for evaluation of shared‐care models, where potential benefits such as reduced travel times and improved continuity may be important to patients but not captured in mortality or adherence data. Further prospective evaluation of ILD services would benefit from standardised collection of adverse events, treatment interruptions, and dose reductions along with longitudinal health‐related quality of life measures, patient‐reported outcome measures and satisfaction metrics, allowing understanding of the patient experience of service delivery.

A further limitation is the potential for selection bias between tiers. In routine practice follow‐up location was primarily determined based on the patient's local respiratory services, rather than clinical severity. Measured baseline demographic, comorbidity, and lung‐function characteristics were similar between tiers, and multivariate analyses did not alter the lack of association between tier status and mortality or adherence; however, given that allocation was not randomised, residual confounding by service‐level factors cannot be excluded. Similarly, only 60 patients were followed up by a Tier 3 site, which may result in outcomes being underpowered, and further work on larger or international cohorts would be useful to further clarify our findings. Finally, because antifibrotic prescribing and access to support were centralised within the network, our findings should be interpreted as an evaluation of supported local follow‐up rather than fully decentralised ILD care.

In conclusion, this retrospective real‐world study found no evidence of differences in clinical outcomes examined based on tier of follow‐up. These findings support the feasibility of delivery of routine ILD follow‐up through a tiered shared‐care model in which local services have continued access to specialist support.

## Author Contributions

All authors contributed to the study conception and design. Data collection and analysis were carried out by Fred Fyles and Alice McKinnon. The first draft of the manuscript was written by Fred Fyles, and all authors commented on the manuscript. All authors read and approved the final manuscript before submission.

## Funding

The authors have nothing to report.

## Ethics Statement

This study was conducted as a service evaluation using routinely collected clinical data. The project was reviewed and approved through local audit and quality improvement governance processes. Formal research ethics committee approval was not required in accordance with UK Health Research Authority guidance.

## Conflicts of Interest

Joanne Sutcliffe reports a relationship with Boehringer Ingelheim Pharmaceuticals Inc. that includes: speaking and lecture fees and travel reimbursement. Lisa G. Spencer (L.G.S.) reports a relationship with Boehringer Ingelheim Pharmaceuticals Inc. that includes: consulting or advisory and speaking and lecture fees. L.G.S. reports a relationship with AstraZeneca Pharmaceuticals LP that includes: speaking and lecture fees. L.G.S. reports a relationship with Chiesi Pharmaceuticals Inc. that includes: travel reimbursement. L.G.S. reports a relationship with Grupo Ferrer Internacional S.A. that includes: consulting or advisory. L.G.S. reports a relationship with Daiichi Sankyo Inc. that includes: consulting or advisory. Sarah Agnew reports a relationship with Boehringer Ingelheim Pharmaceuticals Inc. that includes: consulting or advisory and speaking and lecture fees. Fred Fyles reports a relationship with Konica Minolta Inc. that includes: travel reimbursement. Other authors declare that they have no known competing financial interests or personal relationships that could have appeared to influence the work reported in this paper.

## Supporting information


**Table S1:** Overview of services offered in Merseyside ILD services, with tiers based on Action for Pulmonary Fibrosis descriptions.

**Table S2:** Side effect profiles among patients with ILD prescribed antifibrotics from 01/01/2022 to 31/12/2023, subdivided by antifibrotic choice.

## Data Availability

The datasets generated during the study are not publicly available due to information governance and patient confidentiality requirements. De‐identified data may be made available from the corresponding author upon reasonable request, subject to approval by our Information Governance team.
