# Supplementary material for: Impact of Tiered Service Delivery Model on Antifibrotic Adherence and Outcomes Among Patients With Interstitial Lung Disease
Source: Clin Respir J. 2026 Oct 25;20(10):e70239. doi: 10.1111/crj.70239 (PMC13696157; doi:10.1111/crj.70239)
Supplement: Supplementary file 1 [file CRJ-20-e70239-s001.docx]

| Discontinuation Reason | | Overall (n=275) | Nintedanib (n=186) | Pirfenidone (n=89) | p-value |
| --- | --- | --- | --- | --- | --- |
| Physician-Initiated | | | | | |
|  | Weight Loss, n (%) | 15 (5%) | 11 (6%) | 4 (4%) | 0.825 |
|  | Clinical Deterioration, n (%) | 9 (3%) | 6 (3%) | 3 (3%) | 0.753 |
|  | Deranged LFTs, n (%) | 6 (2%) | 5 (3%) | 1 (1%) | 0.688 |
|  | Medication-related AE, n (%) | 6 (2%) | 1 (1%) | 5 (6%) | 0.025 |
|  | VTE/bleed, n (%) | 6 (2%) | 6 (3%) | 0 (0%) | 0.200 |
|  | Lung transplant, n (%) | 3 (1%) | 3 (2%) | 0 (0%) | 0.554 |
|  | Deranged renal function, n (%) | 2 (1%) | 2 (1%) | 0 (0%) | 0.818 |
| Patient-Initiated | | | | | |
|  | Diarrhoea, n (%) | 39 (14%) | 34 (18%) | 5 (6%) | 0.005 |
|  | Appetite Loss, n (%) | 33 (12%) | 16 (9%) | 17 (19%) | 0.028 |
|  | Nausea/Vomiting, n (%) | 33 (12%) | 18 (10%) | 15 (17%) | 0.162 |
|  | Lethargy/Malaise, n (%) | 21 (8%) | 5 (3%) | 16 (18%) | <0.001 |
|  | Dizziness, n (%) | 6 (2%) | 1 (1%) | 5 (6%) | 0.003 |
|  | Abdominal Pain, n (%) | 6 (2%) | 5 (3%) | 1 (1%) | 0.661 |

Supplementary Table 2: Side effect profiles among patients with ILD prescribed antifibrotics from 01/01/2022-31/12/2023, subdivided by antifibrotic choice
