# Supplementary material for: Impact of Tiered Service Delivery Model on Antifibrotic Adherence and Outcomes Among Patients With Interstitial Lung Disease
Source: Clin Respir J. 2026 Oct 25;20(10):e70239. doi: 10.1111/crj.70239 (PMC13696157; doi:10.1111/crj.70239)
Supplement: Supplementary file 2 [file CRJ-20-e70239-s002.docx]

Supplementary Table 1: Overview of services offered in Merseyside ILD services, with tiers based on Action for Pulmonary Fibrosis descriptions

| Tier | Description | Staffing | Antifibrotic  Prescribing Centre | Roles and responsibilities | Tier 3 support |
| --- | --- | --- | --- | --- | --- |
| Tier 3 | NHSE-commissioned regional specialist ILD service with experience in management of complex ILD and dedicated ILD clinic provision | - Specialist respiratory consultants with expertise in ILD - Specialist ILD nurses - Specialist thoracic radiologists and pathologists | Yes | - Coordinates regional MDT discussion and specialist ILD management - Prescribes and initiates antifibrotic therapy - Provides specialist advice and support to network sites | N/A – regional specialist hub |
| Tier 2 | Dedicated ILD services within the respiratory directorate, supported by specialist input from Tier 3 hub | - Local ILD lead respiratory consultant - Specialised respiratory nurse with ILD expertise including antifibrotic monitoring and adverse effect management | Can be prescribing or non-prescribing; all Merseyside sites were non-prescribing during the study period | - Coordinates local MDT discussion - Provides ongoing local ILD follow-up and monitoring | - Medic-to-medic advice - Virtual MDT support - Specialist ILD nurse and pharmacist support from Tier 3 as required |
| Tier 1 | ILD care delivered within general respiratory clinic, supported by specialist input from Tier 3 hub | - Respiratory consultant without dedicated ILD subspecialist role - General respiratory nursing support | No | - Provides ongoing local ILD follow-up and monitoring - Escalation to specialist services where required | - Medic-to-medic advice - Virtual MDT support - Specialist ILD nurse and pharmacist support from Tier 3 as required |
